# Elevated CO_2_ Can Improve the Tolerance of *Avena sativa* to Cope with Zirconium Pollution by Enhancing ROS Homeostasis

**DOI:** 10.3390/plants12223792

**Published:** 2023-11-07

**Authors:** Mahmoud M. Y. Madany, Hamada AbdElgawad, Doaa A. Galilah, Ahmed M. A. Khalil, Ahmed M. Saleh

**Affiliations:** 1 Biology Department, Faculty of Science, Taibah University, Al-Madinah Al-Munawarah 41411, Saudi Arabia; 2 Botany and Microbiology Department, Faculty of Science, Beni-Suef University, Beni-Suef 62521, Egypt; 3 Botany Department, Faculty of Science, Mansoura University, Mansoura 35516, Egypt; 4 Biology Department, Faculty of Science at Yanbu, Taibah University, King Khalid Rd., Al Amoedi, Yanbu El-Bahr 46423, Saudi Arabia

**Keywords:** antioxidants, ascorbate/glutathione cycle, *Avena sativa*, oxidative damage, toxic metals

## Abstract

Zirconium (Zr) is one of the toxic metals that are heavily incorporated into the ecosystem due to intensive human activities. Their accumulation in the ecosystem disrupts the food chain, causing undesired alterations. Despite Zr’s phytotoxicity, its impact on plant growth and redox status remains unclear, particularly if combined with elevated CO_2_ (eCO_2_). Therefore, a greenhouse pot experiment was conducted to test the hypothesis that eCO_2_ can alleviate the phytotoxic impact of Zr upon oat (*Avena sativa*) plants by enhancing their growth and redox homeostasis. A complete randomized block experimental design (CRBD) was applied to test our hypothesis. Generally, contamination with Zr strikingly diminished the biomass and photosynthetic efficiency of oat plants. Accordingly, contamination with Zr triggered remarkable oxidative damage in oat plants, with concomitant alteration in the antioxidant defense system of oat plants. Contrarily, elevated levels of CO_2_ (eCO_2_) significantly mitigated the adverse effect of Zr upon both fresh and dry weights as well as the photosynthesis of oat plants. The improved photosynthesis consequently quenched the oxidative damage caused by Zr by reducing the levels of both H_2_O_2_ and MDA. Moreover, eCO_2_ augmented the total antioxidant capacity with the concomitant accumulation of molecular antioxidants (e.g., polyphenols, flavonoids). In addition, eCO_2_ not only improved the activities of antioxidant enzymes such as peroxidase (POX), superoxide dismutase (SOD) and catalase (CAT) but also boosted the ASC/GSH metabolic pool that plays a pivotal role in regulating redox homeostasis in plant cells. In this regard, our research offers a novel perspective by delving into the previously unexplored realm of the alleviative effects of eCO_2_. It sheds light on how eCO_2_ distinctively mitigates oxidative stress induced by Zr, achieving this by orchestrating adjustments to the redox balance within oat plants.

## 1. Introduction

Growing environmental and health apprehensions have been raised regarding toxic metals, primarily due to their extensive dissemination throughout the ecosystem. The accumulation of such toxic metals in the ecosystem imposes rigorous imperilment on both human health and agricultural productivity. There are several reasons for the introduction of such toxic metals to the environment. Anthropogenic endeavors such as mining activities, the application of pesticides and industrial wastes are the common sources of such toxic metals [1]. Once they have been introduced to the ecosystem, they are incorporated into the food chain, causing undesired alterations [2]. Although plants need some of these metals for their growth and development, the existence of these metals in high levels causes a tremendous disruption in plant physiology and metabolism [3]. Like other toxic metals, Zirconium (Zr) is widely distributed in the Earth’s crust [4]. It is considered a reactive metal but is more resistant to corrosion than many other metals. This is due to the formation of a thin layer of zirconium dioxide (ZrO_2_, also known as zirconia) that acts as a protective layer for the underlying metal against further oxidation [5]. Zr and its alloys are widely used in different human activities. It is commonly applied in the nuclear industry due to its high resistance to corrosion, in addition to being used as cladding for nuclear fuel rods in reactors [5]. Furthermore, Zr compounds can be utilized in various sectors of industries such as ceramics, refractories, and electronics. More interestingly, ZrO_2_ is a material that can be utilized in various applications, including dental implants, catalysts, and solid oxide fuel cells. The widespread occurrence of Zr in various activities has led to its extensive presence in our surroundings. This situation has raised alarm within the scientific community due to the potential detrimental effects it poses on human health and agricultural productivity. Zr and its compounds are toxic to human health, so proper handling and safety precautions should be taken to prevent its dispersion [6]. Concerning plants, although Zr is not an essential element for plant growth, excessive exposure to Zr and its compounds may slow plant growth and development and change the soil structure [7]. Nonetheless, when it comes to understanding the effects of Zr on plants, there has been relatively limited research compared to more extensively studied elements. The excessive buildup of Zr within plant tissues has the potential to interfere with nutrient absorption and crucial metabolic processes, ultimately resulting in a significant decline in plant growth [6]. Shaid et al. [6] also declared that Zr can interfere with the availability of some essential nutrients, such as Ca, Mg, and K which are vital for most plant metabolic processes, something that can hinder overall plant growth and development. Moreover, excessive exposure to Zr has been reported to alter the photosynthetic pigments of *Chlorella pyrenoidosa*, leading to a negative impact on culture mass and yield [8]. Both root function and development can also be affected by higher exposure to Zr. The disturbance in root function and growth, in turn, can impact the plant’s capacity to absorb nutrients and water, diminishing its ability to acquire vital resources, leading to stunted growth and decreased vitality [9]. Furthermore, Fodor et al. [9] declared that plant exposure to Zr may trigger plant oxidative stress that causes an imbalance between the production of ROS and the ability to detoxify them. This oxidative damage can disrupt the cellular components and slow different metabolic pathways in plants [10]. It is crucial to understand that the detrimental impacts of Zr on plants can be influenced by various factors, including the type and the concentration of Zr in the soil, soil conditions and the specific plant species involved. Nonetheless, the precise mechanisms through which Zr influences plant metabolism remain incompletely elucidated, underscoring the need for further research to comprehensively assess the impact on plant growth and metabolic processes. To this end, researchers should explore environmentally friendly and economically viable agricultural approaches to mitigate the potential harmful effects of excessive Zr accumulation. One of these tools is by enriching the atmosphere of plants with carbon dioxide. 

The intensive increase in atmospheric CO_2_ levels is a major environmental challenge. This elevation is expected to further modify soil properties, altering the growth and development of economically important crops. Progressive industrial activities have caused a rapid increase in CO_2_ concentration from 280 ppm to 400 ppm [11]. Prognostications have shown that this increment will continue to increase as a consequence of immense human industrial impact [12]. In point of fact, elevation in CO_2_ within a physiological range has been proven to enrich plant growth, enhancing photosynthetic carbon metabolism with consequent boosting in assimilation [10]. The enhancement in carbon metabolism will accordingly be reflected in the partitioning of plant carbon and nitrogen [11]. Furthermore, several studies showed that eCO_2_ could alleviate the detrimental effects of a variety of environmental constraints upon plant growth and metabolism [10,13,14]. This potential of eCO_2_ is attributed to its effective changes in stomatal conductance, which play a crucial role in enhancing plant water uptake [15]. More interestingly, eCO_2_ could mitigate the imposed stress by modulating the redox homeostasis through regulating ROS production and scavenging [16]. Therefore, the scientific community should pay attention to the impact of eCO_2_ in boosting plant tolerance under different environmental challenges, especially for economically important crops such as oat plants. In this regard, eCO_2_ raised the tolerance level of two barley cultivars grown under salinity stress [17]. Furthermore, Mhamdi and Noctor [18] suggested that Arabidopsis subjected to eCO_2_ treatment exhibits increased resistance against bacterial and fungal invasions. Likewise, it was found that treatment with eCO_2_ enhanced the defense system of Arabidopsis against different leaf and root pathogenic fungi [19]. Furthermore, tomato plants treated with eCO_2_ can cope with heat stress by enhancing photosynthesis and redox homeostasis [20]. Overall, these investigations and more were directed to study the combined effect of both eCO_2_ and different biotic and abiotic stresses. Nevertheless, the effect of raising atmospheric CO_2_ upon a toxic metal like Zr has not yet been considered. Therefore, our study was conducted to closely investigate the impact of two levels of CO_2_ (ambient, 420 ppm, and elevated, 710 ppm) on the growth and redox status of oat (*Avena sativa*) grown under the curb of Zr pollution. We further aim to verify the hypothesis that eCO_2_ amplifies carbohydrate portioning by bolstering photosynthetic efficiency. This, in turn, fortifies the antioxidant defense system, enabling it to counteract the oxidative damage caused by Zr. 

## 2. Material and Methods

### 2.1. Plant Materials and Treatment 

In order to gain a deeper insight into the impact of Zr on the plants, this study was performed with conditions that approached environmentally realistic conditions that closely mimic the environmental scenarios. The seeds of *Avena sativa* were homogeneously selected and sterilized with Na-hypochlorite (5% *v/v*, 20 min) and then washed thoroughly with distilled water. The sterilized seeds were sown in 20 cm diameter, 30 cm height polyvinyl chloride (PVC) tubes containing sandy soil with adjusted pH (7.6). The soil was irrigated daily to maintain soil capacity at 68%. The cultivation of plants took place within chambers that were illuminated by sunlight and maintained under controlled temperature and CO_2_ levels. The upper parts of these sunlit chambers were constructed with a transparent polycarbonate plate to permit light entry. The temperature was set to 26 °C during the day and 20 °C at night, and Photosynthetic Active Radiation was detected using an SDEC, type JYP1000 quantum sensor. The pots were divided into four groups, each with 4 replicates: (1) ambient CO_2_ (aCO_2_, 420 ppm); (2) aCO_2_ + Zr (400 mg/kg soil); (3) future climate CO_2_ (eCO_2_, 710 ppm); and (4) eCO_2_ + Zr (400 mg/kg soil). Zr was introduced to the soil as ZrSO_4_.4H_2_O (Sigma-Aldrich, Germany). The soil was consistently mixed with the desired amount of Zr. After 8 weeks, the rhizosphere soil as well as plant tissues were collected and kept in −80 °C for further biological analyses. The fresh and dry weights of oat plants were recorded. 

### 2.2. Determination of Zr in Plant Tissues 

To extract Zr, the plant samples, which had been dried at 70 °C, were subjected to a treatment involving 13 M nitric acid at a temperature of 185 °C for a duration of 25 min. Subsequently, the concentration of elements was determined using a quadrupole inductively coupled plasma mass spectrometry (ICP-MS; model 820-MS) setup, linked with a glass nebulizer operating at a flow rate of 0.4 mL/min. To generate calibration curves, external standards were prepared at concentrations ranging from 1 to 600 μg/L. Additionally, yttrium was included as an internal standard during the extraction process to calibrate the efficiency of the nebulizer. Standard mineral samples were prepared using 0.23 M nitric acid.

### 2.3. Photosynthesis and Photosynthetic Related Parameters

Photosynthetic and stomatal conductance measurements were carried out following the methodologies detailed in Ainsworht and Rogers [15]. To determine photosynthesis under saturating light conditions (Asat, μmol CO_2_ m^−2^ s^−1^), a LI-COR LI-6400 instrument (LI-COR Inc., Lincoln, NE, USA) was used. Each step of the measurement included a leaf equilibration period of at least 5 min before recording data. The LI-COR leaf chamber was configured with conditions set at either 400 or 620 ppm CO_2_, a block temperature of 22 °C, and a saturated photon flux density of 1500 μmol m^−2^ s^−1^. Stomatal conductance (gs, mol CO_2_ m^−2^ s^−1^) was assessed on the abaxial surface of fully developed leaves utilizing a leaf porometer (Model SC-1, Decagon Devices, Inc., Hopkins, Pullman, WA, USA). The average vapor pressure deficit and leaf temperature were maintained at 0.37 ± 0.02 and 20 ± 2.02, respectively. Chlorophyll fluorescence was gauged on fully expanded leaves that had been dark-acclimated, using an FMS-2 pulse-modulated fluorometer (Hansatech Instruments, Norfolk, UK). For leaves adapted to darkness for 30 min, minimal fluorescence (F0) and maximal fluorescence (Fm) were recorded. The photochemical efficiency of Photosystem II (PSII) (Fv/Fm) for these dark-adapted leaves was computed, where Fv (maximal variable fluorescence) was calculated as Fm−F0. Chlorophyll a, chlorophyll b and carotenoid concentrations were assessed in oat shoots homogenized using acetone [21]. RuBisCo activity was quantified using a non-radioactive microplate-based method that involved the enzymatic cycle between glycerol-3-phosphate dehydrogenase and glycerol-3-phosphate oxidase to measure the product, 3-phosphoglycerate (3-PGA) [22].

### 2.4. Determination of Oxidative Stress Markers 

Lipid peroxidation was assessed using the thiobarbituric acid–malondialdehyde (TBA–MDA) assay according to the method outlined by Senthilkumar et al. [23]. For this, 0.3 g of frozen oat tissue was homogenized in 80% ethanol using a mortar and pestle. Centrifuge the extract (10,000× *g* for 10 min). Take 0.5 mL of the clear supernatant then add 2.5 mL of the TBA and 0.5 mL butylated hydroxytoluene (BHT) to prevent further oxidation during heating. Incubate in a boiling water bath for 30 min, centrifuge and measure the absorbance at 532 nm. MDA concentration was calculated using the molar extinction coefficient of MDA (1.53 mM^−1^cm^−1^). The quantification of H_2_O_2_ levels was performed using the xylenol orange-based FOX1 technique [22]. Mix 0.1 mL of plant extract with 0.9 mL FOX1 reagent (4.4 mM xylenol orange, 2.6 mM sorbitol, 25 mM sulfuric acid, and 0.2 mM ferrous ammonium sulfate) then add 0.05 mL of ascorbic acid. Incubate the mixture at room temperature for 30 min in the dark, then centrifuge (10,000× *g* for 5 min). Measure the absorbance of the colored complex at 560 nm. H_2_O_2_ levels were calculated using a standard curve with known concentrations of H_2_O_2_.

### 2.5. Determination of Antioxidants’ Secondary Metabolites 

Polyphenols and flavonoids were measured employing the Folin–Ciocalteu and aluminum chloride assays, respectively, as outlined in the reference [24]. The separation and quantification of tocopherols were achieved through High-Performance Liquid Chromatography (HPLC) using normal phase conditions with a Shimadzu system based in Hertogenbosch, Netherlands. The HPLC setup involved a Particil Pac column (5 μm column material, length 250 mm, i.d. 4.6 mm). Total Antioxidant Capacity (TAC) was evaluated following the Ferric Reducing Antioxidant Power (FRAP) method described by Benzie and Strain [25], wherein Trolox (Sigma-Aldrich, St. Louis, MO, USA) was employed as an internal standard. 

### 2.6. Estimation of Enzymatic Antioxidants 

For the determination of antioxidant enzyme activity, proteins were extracted using K-phosphate extraction buffer (50 mM, pH 7.0) supplemented with 10% PVPP (*w/v*), 0.25% Triton X-100 (*v/v*) and 1 mM PMSF. Peroxidase (POX) activity was assessed through the oxidation of pyrogallol at 430 nm [26], while superoxide dismutase (SOD) enzyme activities were determined by monitoring the inhibition of NBT reduction at 560 nm [27]. A known weight of frozen tissue (0.5 g) was homogenized in 1 ml extraction buffer (0.1 M phosphate buffer, pH 6.0), then centrifuged (4000× *g*, for 10 min at 4 °C). Then, 1 ml clear extract was taken and mixed with 1 ml assay mixture (20 M pyrogallol; 20 mM guaiacol; 30% H_2_O_2_). After mixing well, the produced color was measured at 420 nm. Dehydroascorbate reductase (DHAR), glutathione reductase (GR), ascorbate peroxidase (APX), and monodehydroascorbate reductase (MDHAR) were evaluated spectrophotometrically based on the method described by Murshed et al., [28] by utilizing 0.05 M MES/KOH as the buffer. Catalase (CAT) activity was quantified by observing the rate of H_2_O_2_ decomposition at 240 nm [29]. A known fresh weight of plant tissue was homogenized in 0.1 M potassium phosphate buffer (pH 7.0), then centrifuged at low speed (3000 g) for 10 min at 4 °C. Then, 100 µL clear extract was mixed with 2 mL assay mixture (0.1 M KH_2_PO_4_ buffer, pH 7.0; 30% H_2_O_2_). The change in color at 240 nm was measured over time. Glutathione peroxidase (GPX) activity was measured by monitoring the reduction of NADPH at 340 nm [30]. For GPX activity, a known weight of powdered fresh tissue was homogenized in ice-cold 0.1 M KH_2_PO_4_ buffer (pH 7.0). After centrifugation, 50 µL of the clear extract was mixed with the assay mixture (0.1 M KH_2_PO_4;_ 1 M GSH; 1 mM NADPH). The change in color was monitored at 340 nm, after adding 30% H_2_O_2_ to initiate the reaction. The total soluble protein concentration was determined using the Lowry technique [31].

### 2.7. Determination of AsA/GSH Metabolites 

Reduced ascorbate (ASC) and reduced glutathione (GSH) levels were measured using High-Performance Liquid Chromatography (HPLC) with a Shimadzu SIL10-ADvp system and a C18 column (Spherisorb ODS2, 5 μm particle diameter, 4.6 × 250 mm, Waters). The concentrations of total ascorbate (ASC + DHA) and glutathione (GSH + GSSG) were determined after reduction with dithiothreitol (DTT), following the methodology outlined by Sinha et al. [32]. Enzyme activities including superoxide dismutase (SOD), glutathione peroxidase (GPX), ascorbate peroxidase (APX), glutathione reductase (GR), monodehydroascorbate reductase (MDHAR), dehydroascorbate reductase (DHAR), and glutathione-S-transferase (GST) were assessed according to the procedures detailed in our previous research [33].

### 2.8. Statistical Analysis 

The outcomes of the measured parameters are presented as the average of four biological replicates (n = 4). Statistical evaluation was executed utilizing one-way ANOVA and two-way ANOVA within the SPSS 21 software (Inc., Chicago, IL, USA). Mean distinctions were ascertained through Fisher’s least significance difference (LSD) test. The normality of data distribution and the equality of variances were assessed using the Kolmogorov–Smirnov test and Levene’s test, respectively. Furthermore, a comparative evaluation of pollution severity between Zr and Zr + eCO_2_ was conducted through LSD, with a significance threshold set at *p* < 0.05.

## 3. Results 

### 3.1. Effect of eCO_2_ on Zr Cumulation and the Growth of Oat Plants 

**Figure 1:**
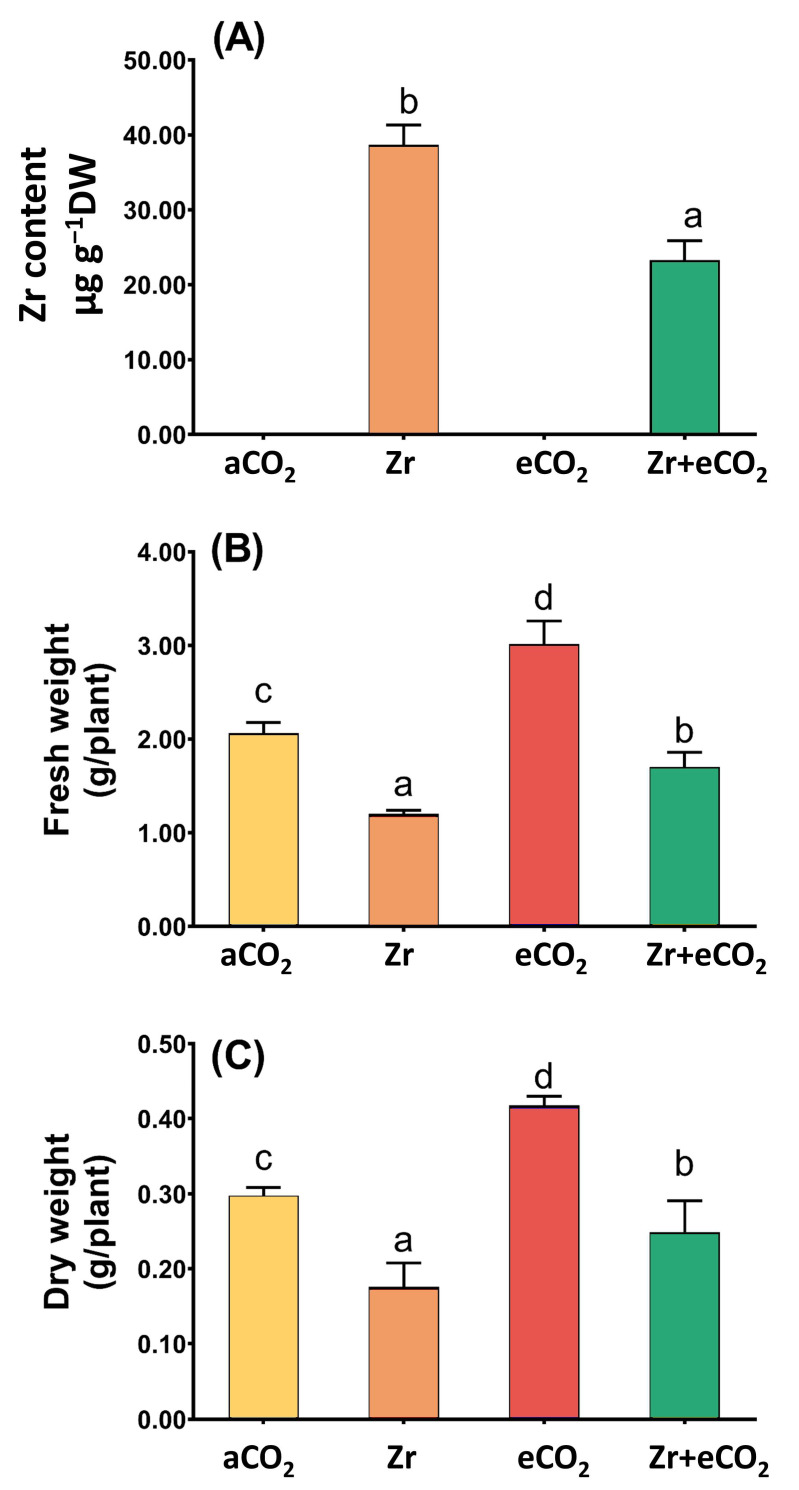
The combined effect of both eCO_2_ and/or Zr upon (**A**) Zr accumulation, (**B**) fresh weight and (**C**) dry weight of oat plants. Four biological replicates were used to investigate the response. The vertical error bars are the standard error (SE). Fisher’s LSD test (*p* < 0.05, n = 4) was used for pairwise comparison between groups. The different letters indicate significant differences between the means of each group.

Our results showed that the levels of Zr in contaminated oat plants was very high; however, the cotreatment of oat plants with both Zr and elevated levels of eCO_2_ caused a remarkable reduction in the levels of Zr (~40% reduction) when compared with oat plants grown in contaminated soils (Figure 1A). This significant reduction in the levels of Zr due to the application of eCO_2_ prompted us to measure other growth parameters stand on the mitigative impact of eCO_2_. In this regard, oat plants experienced a noticeable reduction in the biomass (fresh and dry weights) in response to Zr pollution when compared with uncontaminated control plants (Figure 1B,C). On the other hand, treatment with eCO_2_ significantly enriched the biomass of oat plants (*p* < 0.05) as compared with normal control plants. Further, the co-application of both Zr and CO_2_ noticeably enhanced both fresh and dry weights of oat relative to plants contaminated with Zr. A two-way ANOVA revealed that both Zr and eCO_2_ significantly interacted to affect both fresh (*p* < 0.001) and dry (*p* < 0.05) weights of oat plans (Table 1). 

### 3.2. Photosynthesis of Oats as Affected by Zr and/or eCO_2_

**Figure 2:**
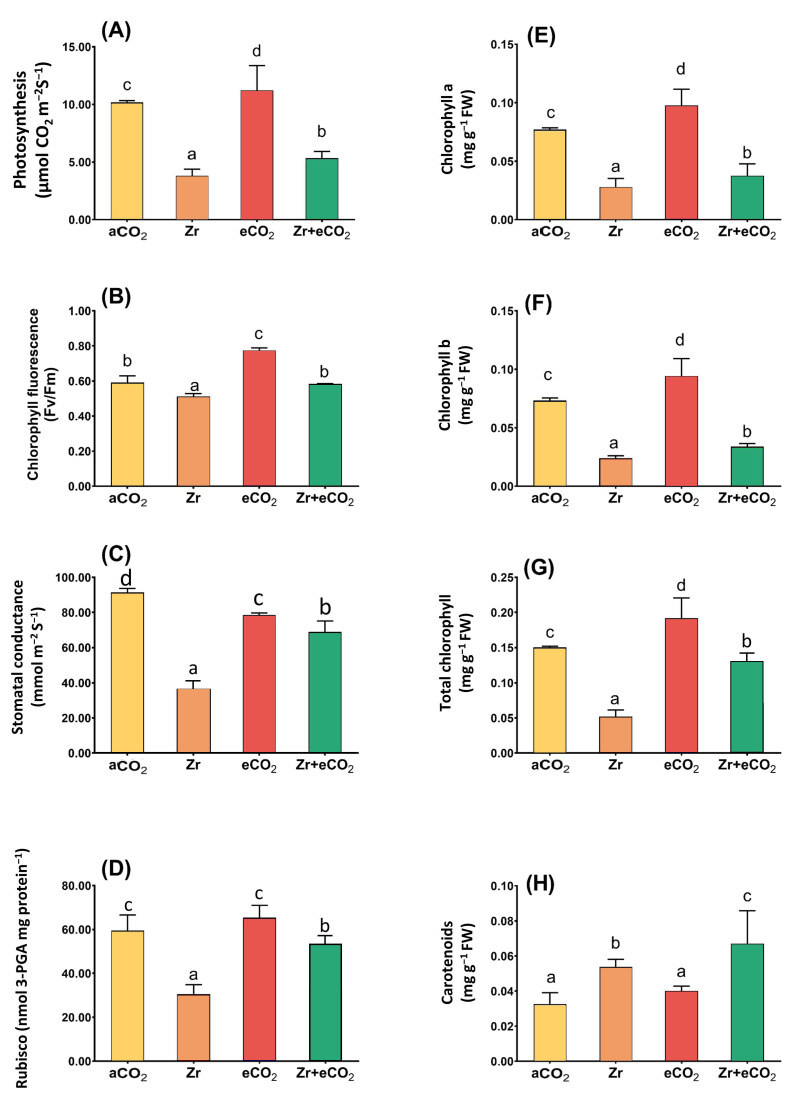
The combined effect of eCO_2_ and/or Zr upon photosynthetic efficiency of oat plants. (**A**) rate of photosynthesis, (**B**) chlorophyll fluorescence, (**C**) stomatal conductance, (**D**) RuBisco activity, (**E**) chlorophyll a, (**F**) chlorophyll b, (**G**) total chlorophyll, and (**H**) carotenoids. Four biological replicates were used to investigate the response. The vertical error bars are the standard error (SE). Fisher’s LSD test (*p* < 0.05, n = 4) was used for pairwise comparison between groups. The different letters indicate significant differences between the means of each group.

The enrichment in oat growth due to the application of eCO_2_ in combination with Zr encouraged us to investigate its effect upon the photosynthetic efficiency (Figure 2). As intended, the delay in the biomass of oat in response to Zr pollution was associated with a concomitant reduction in the photosynthetic machinery. This reduction was more obvious in the levels of Chl a, Chl b and Chl (a + b), and accordingly the rate of photosynthesis, which decreased by 61%, 71%, 50%, and 62%, respectively, in contaminated oat as compared with uncontaminated control plants (Figure 2A,E–G). This could be attributed to the significant decline in both stomatal and non-stomatal parameters such as stomatal conductance and RuBisco that exhibited about 50% reduction relative to uncontaminated control plants (Figure 2C,D). On the other hand, the individual treatment with eCO_2_ significantly affected the rate of photosynthesis in oat plants. More interestingly, the application of eCO_2_ along with Zr recovered the adverse effect of Zr upon photosynthetic parameters. For instance, stomatal conductance and RuBisco increased by about 60% and 40%, respectively, when compared with oat grown under aCO_2_ and Zr conditions (Figure 2C,D). More obviously, total chlorophyll content experienced a three-fold increase relative to plants grown in aCO_2_ and Zr conditions (Figure 2G). The two-way ANOVA showed different interactive responses between the two independent variables (eCO_2_ and Zr) for all photosynthetic parameters (Table 1). The co-application of eCO_2_ and Zr (eCO_2_ + Zr) significantly enhanced the chlorophyll (a + b) and carotenoids (*p* < 0.05) as well as chlorophyll fluorescence (*p* < 0.001).

### 3.3. Influence of eCO_2_ on the Zr-Induced Oxidative Damage in Oat Plants

**Figure 3:**
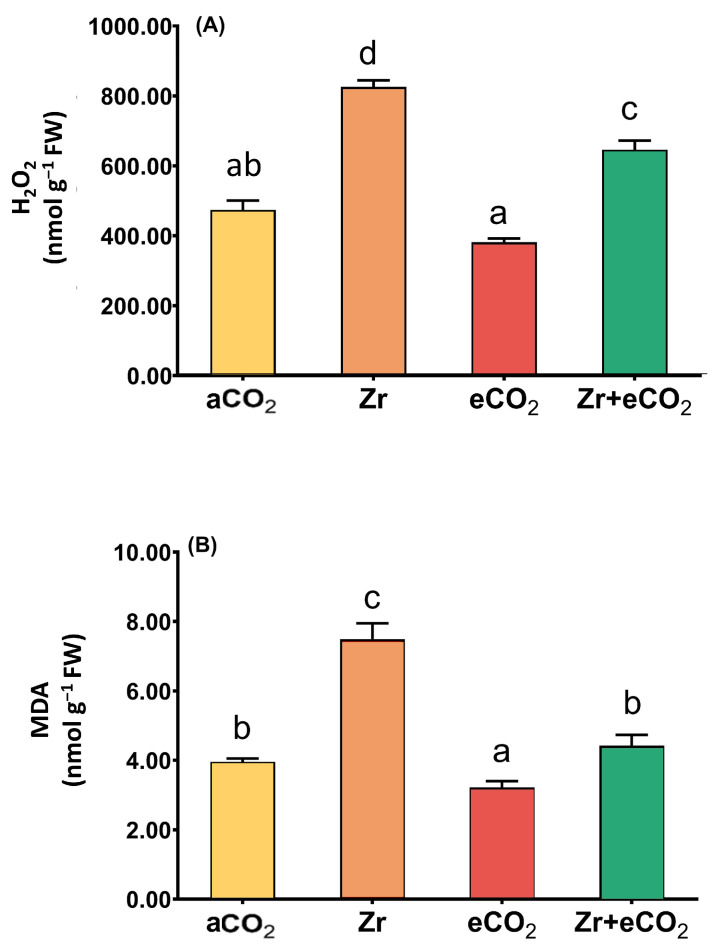
The combined effect of eCO_2_ and/or Zr on the levels of oxidative stress markers of oat plants. (**A**) hydrogen peroxide (H2O2) and (**B**) malondialdehyde (MDA). Four biological replicates are used to investigate the response. The vertical error bars are the standard error (SE). Fisher’s LSD test (*p* < 0.05, n = 4) was used for pairwise comparison between groups. The different letters indicate significant differences between the means of each group.

The accumulation of lipid peroxidation (MDA) and H_2_O_2_ is considered one of the main markers for oxidative damage in plants [34]. In this context, the individual application of Zr strikingly increased the levels of H_2_O_2_ (a two-fold increase) in oat plants (Figure 3A). It is likely that the levels of MDA exhibited a noticeable accumulation in oat plants in response to treatment with Zr (87% increase) relative to plants grown in eCO_2_-uncontaminated conditions. Additionally, individual treatment with eCO_2_ reduced the levels of both H_2_O_2_ and MDA a little bit. The results showed that eCO_2_ alone reduced the levels of H_2_O_2_ and MDA in oat by about 15% and 25%, respectively, as compared with the counter control plants treated with aCO_2_ (Figure 3). More interestingly, the application of eCO_2_ in combination with Zr caused a tremendous reduction in the levels of H_2_O_2_ and MDA. The reduction was more pronounced in the levels of MDA that exhibited a 47% reduction relative to Zr-treated control plants (Figure 3B). Overall, a significant interaction was found between eCO_2_ and Zr for both H_2_O_2_ and MDA (*p* < 0.01 and *p* < 0.001, respectively) (Table 2).

### 3.4. eCO_2_ and Non-Enzymatic Antioxidants in Zr-Contaminated Oat Plants

**Figure 4:**
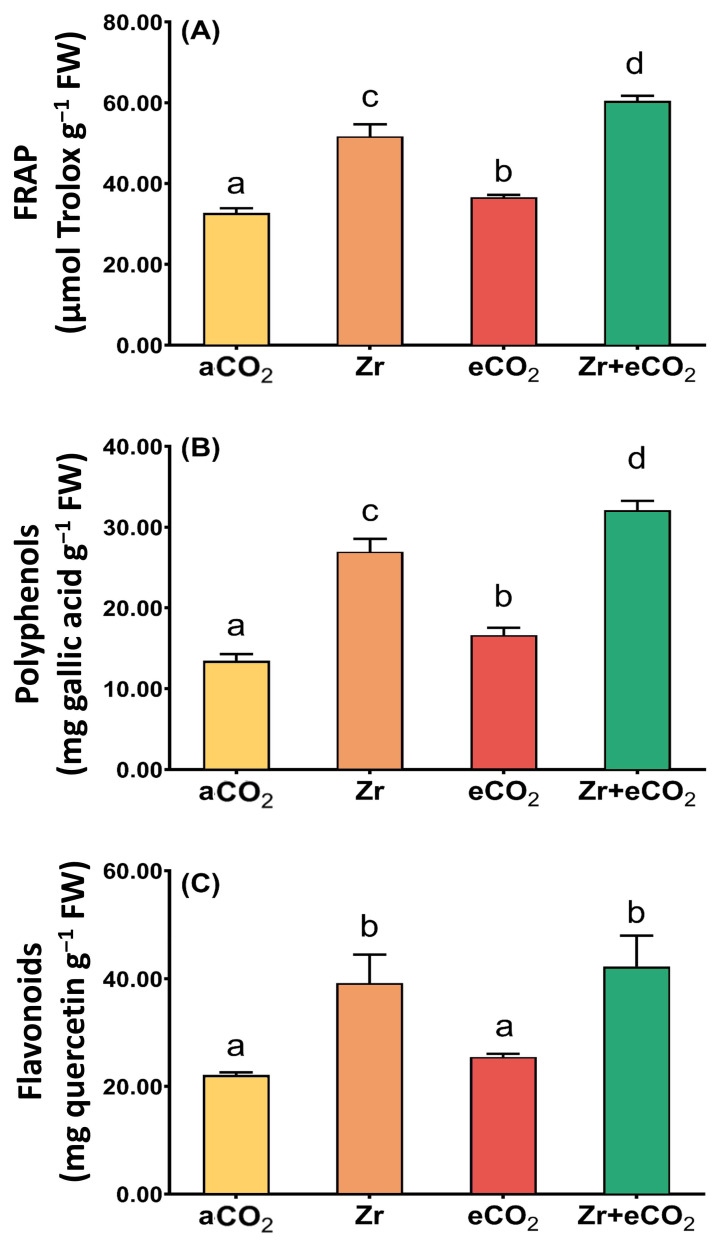
The combined effect of eCO_2_ and/or Zr upon the levels of total antioxidant capacity and molecular antioxidants of oat plants. (**A**) total antioxidant capacity (FRAP), (**B**) polyphenols, (**C**) flavonoids. Four biological replicates were used to investigate the response. The vertical error bars are the standard error (SE). Fisher’s LSD test (*p* < 0.05, n = 4) was used for pairwise comparison between groups. The different letters indicate significant differences between the means of each group.

To contend with the oxidative damage, plants augmented their antioxidant defense arsenal to keep cell viability against the phytotoxic impact of Zr. Therefore, we aimed to measure the total antioxidant capacity (FRAP) as well as some non-enzymatic antioxidants (polyphenols and flavonoids) in oat plants treated with eCO_2_ and/or Zr (Figure 4). Our results revealed that Zr significantly increased the levels of FRAP in oat (~36% increase) compared to aCO_2_-treated oat plants (Figure 4A). This increment was associated with a remarkable increase in the levels of both polyphenols and flavonoids. Polyphenols experienced a two-fold increase in response to individual treatment with Zr relative to aCO_2_-treated control plants (Figure 4B). Similarly, treatment with Zr increased the levels of flavonoids of oat plants by about 72% as compared with aCO_2_-treated control plants (Figure 4C). Except for flavonoids, eCO_2_ caused a significant elevation in FRAP as well as polyphenols (Figure 4A,B). More interestingly, the application of both Zr and eCO_2_ FRAP caused a two-fold increase in both FRAP and flavonoids of oat plants relative to those grown under aCO_2_. To a greater extent, polyphenols exhibited about a three-fold increment in oat plants grown under both Zr and eCO_2_ if they were compared with those grown under aCO_2_. According to the two-way ANOVA, the interaction between Zr and eCO_2_ had varying significance on the responses. The significance of interaction between factors in polyphenols was higher (*p* < 0.001) than that of both FRAP and flavonoids (*p* < 0.05) (Table 2).

### 3.5. eCO_2_ Affecting the Enzymatic Antioxidants of Zr-Contaminated Oat Plants 

**Figure 5:**
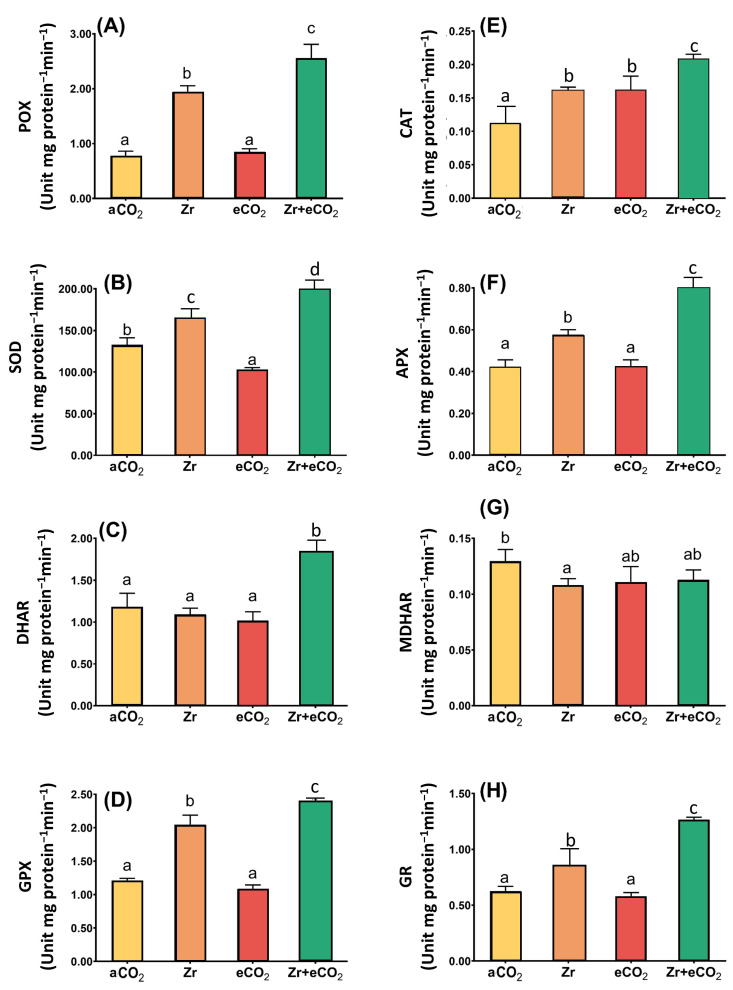
The combined effect of eCO_2_ and/or Zr upon the activities of antioxidant oat plants. (**A**) peroxidase, (**B**) superoxide dismutase, (**C**) dehydroascorbate reductase, (**D**) glutathione peroxidase, (**E**) catalase, (**F**) ascorbate peroxidase, (**G**) monodehydroascorbate reductase, and (**H**) glutathione reductase. Four biological replicates were used to investigate the response. The vertical error bars are the standard error (SE). Fisher’s LSD test (*p* < 0.05, n = 4) was used for pairwise comparison between groups. The different letters indicate significant differences between the means of each group.

Antioxidant enzymes with those of the ASC/GSH cycle play a pivotal role in ROS homeostasis. In our study, we were concerned with investigating the activities of peroxidase (POX), superoxide dismutase (SOD), GSH-peroxidase (GPX) and catalase (CAT), in addition to the enzymes involved in ASC/GSH metabolism in oat plants treated with Zr and eCO_2_, either alone or in combination (Figure 5). Although the treatment of oat plants with Zr alone had no significant effect upon DHAR, and MDHAR, it caused a slight increase in the activities of some other enzymes. For example, SOD activity increased by 39% in response to the treatment of oats with Zr (Figure 5B). Moreover, the application of Zr upon oat plants increased the activity of both CAT and GPX up to 45% and 54%, respectively, when compared with control (aCO_2_) uncontaminated plants (Figure 5D). It is likely that the activity of glutathione reductase (GR) in oat plants exhibited a 33% increase due to the contamination with Zr if compared with plants grown under aCO_2_ conditions (Figure 5H). To a greater extent, POX activity showed an about two-fold increment in response to Zr treatment relative to control plants grown under aCO_2_ conditions (Figure 5A). Compared to aCO_2_, eCO_2_ had no significant impact upon the activities of the enzymatic antioxidants, except for CAT, whose activity increased by 46% relative to plants grown under aCO_2_ conditions (Figure 5E). Contrarily, eCO_2_ highly enhanced the enzymatic antioxidants in oat plants grown in Zr pollution conditions. In this regard, the activities of SOD and DHAR were augmented (30% and 50% increase, respectively) in oat plants in response to the co-application of both eCO_2_ and Zr if compared with aCO_2_-uncontaminated control plants (Figure 5B,C). A further increase was observed in the activities of both GPX and CAT (91% increase for each) due to the treatment of oat with both eCO_2_ and Zr (Figure 5D,E). Additionally, the treatment of oat plants with both eCO_2_ and Zr caused a two-fold enhancement in the activities of both APX and GR in comparison with aCO_2_ uncontaminated control plants (Figure 5F,H). More interestingly, POX activity was increased by about four-fold in oat plants treated with eCO_2_ and Zr relative to aCO_2_-uncontaminated control plants (Figure 5A). A two-way ANOVA declared that a differential interactive effect existed between the two independent variables (Zr and eCO_2_) upon SOD (*p* < 0.001), DHAR (*p* < 0.01) and GPX (*p* < 0.05) (Table 2).

### 3.6. Effect of Zr and/or eCO_2_ on the AsA/GSH Metabolic Pool of Oat Plants

**Figure 6:**
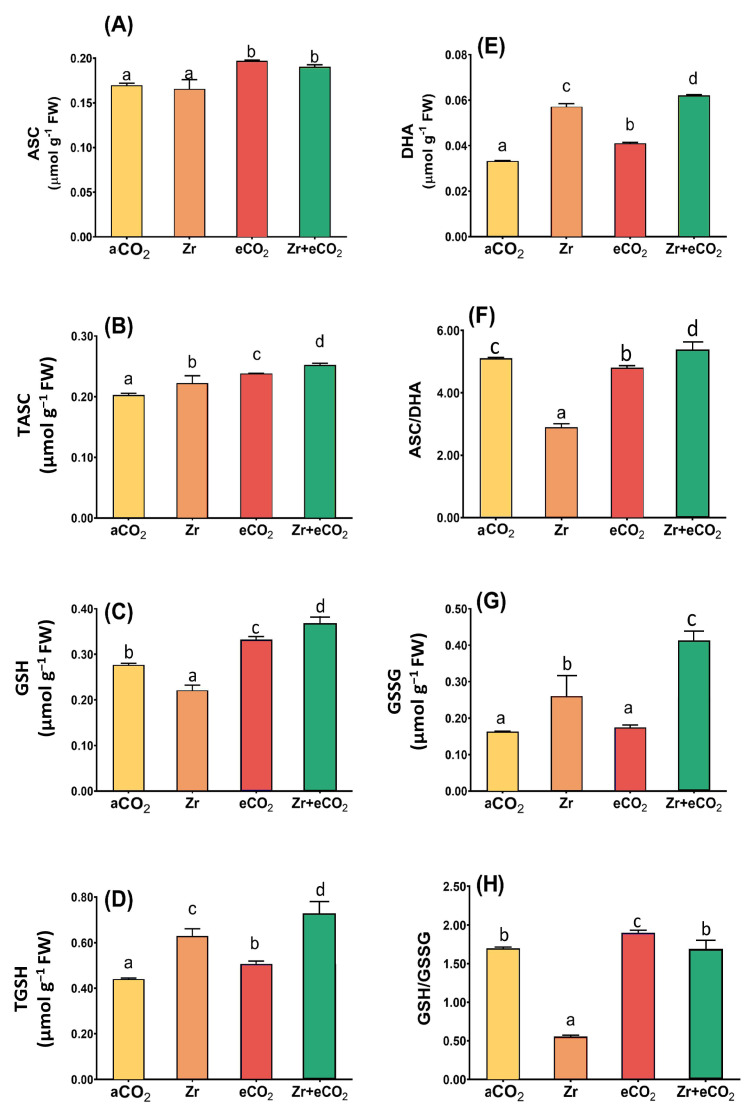
The combined effect of eCO_2_ and/or Zr upon the ascorbate/glutathione metabolic pool of oat plants. (**A**) ascorbic acid, (**B**) total ascorbate, (**C**) glutathione, (**D**) total glutathione, (**E**) dehydroascorbate, (**F**) ascorbate/dehydroascorbate ratio, (**G**) glutathione disulfide and (**H**) glutathione/glutathione disulfide ratio. Four biological replicates were used to investigate the response. The vertical error bars are the standard error (SE). Fisher’s LSD test (*p* < 0.05, n = 4) was used for pairwise comparison between groups. The different letters indicate significant differences between the means of each group.

The ascorbate/glutathione metabolic pathway is involved in different plant metabolic reactions, including those elicited by environmental cues. Therefore, measuring its metabolic components is of great importance for the effectiveness of eCO_2_ in mitigating the phytotoxic effect of Zr. Although Zr had no significant effect on the levels of ascorbate (Figure 6A), it greatly reduced the glutathione levels in oat plants (Figure 6C). In addition, Zr caused a three-fold reduction in the GSH/GSSG ratio in polluted oat relative to aCO_2_-unpolluted control plants (Figure 6H). Contrarily, oat plants grown in soils polluted with Zr exhibited a noticeable increment in the levels of tASC and tGSH (Figure 6B,D). A further elevation was observed in the levels of DHA and GSSG, which increased by 65% and 59% higher than aCO_2_-uncontaminated control plants (Figure 6E,G). Moreover, GSSG experienced a more than two-fold increase in oats due to contamination with Zr that could explain the remarkable reduction in GSH/GSSG ratio (Figure 6G,H). Except for the GSSG and ASC/DHA ratio, the individual application of eCO_2_ significantly enhanced the components of ascorbate/glutathione metabolic pathway in oat plants relative to aCO_2_ control plants (Figure 6). For instance, tGSH showed a 25% increase in oats treated with eCO_2_ alone when compared with aCO_2_ control plants (Figure 6C). More interestingly, eCO_2_ further enhanced the levels of ascorbate/glutathione metabolites in oats when grown under Zr-contamination conditions. In this context, the treatment of oat plants with both eCO_2_ and Zr enhanced the levels of tGSH by about 28% higher than those treated with Zr alone (Figure 6D). Moreover, eCO_2_ improved GSH and DHA by about 66% and 65% in oat plants grown in Zr compared with those grown in Zr alone (Figure 6C,E). A further increase was observed in GSSG, as well as in the ratio of both ASC/DHA and GSH/GSSG (two-fold and three-fold increase, respectively) in response to the treatment of oat plants with both eCO_2_ and Zr, as compared with those treated with Zr alone (Figure 6F–H). Two-way ANOVA indicates presence of interactive effect of both Zr contamination and eCO_2_ treatment that was apparent on the activities of DHA, GSH, TGSH (*p* < 0.05, 0.01, 0.001), in addition to the levels of GSH and GSSG (*p* < 0.01, 0.001) (Table 3). 

## 4. Discussion 

Upon being incorporated into the food chain, Zr becomes a threat to human health as it can be easily taken in by the human body through food or water. Concerning plants, Zr was found to inhibit the germination of seeds and the growth of plant shoots and roots [35]. Nevertheless, there has been a lack of information about its impact upon plant physiology and metabolism. Therefore, our study aims to investigate the phytotoxic impact of Zr upon the growth, photosynthesis and ROS homeostasis of oat plants (*Avena sativa*) and how elevated CO_2_ could ameliorate this phytotoxic effect. 

### 4.1. eCO_2_ Ameliorated the Growth Reduction and Oxidative Burst in Oat That Was Initiated by Zr

Our results showed a remarkable reduction in the growth as well as photosynthesis of oat plants in response to contamination with Zr. These findings highlight the phytotoxic impact of Zr upon plant growth and development. Comparable findings were noted in the case of algae by Simon et al. [8], who reported an adverse impact of Zr on the growth and photosynthesis of *Chlorella pyrenoidosa*. Moreover, the dry matter of maize shoots and roots were dramatically decreased by increasing the concentration of Zr [35]. It is likely that treatment with Zr not only inhibits the germination of *Triticum aestivum* but also adversely inhibits the growth of their shoots and roots [9]. Generally, soil contamination with toxic metals adversely delays the growth and development of plants. In this context, the growth of *Oryza sativa* was highly delayed when grown in soils contaminated with Indium [36]. A similar observation was opined by Kopittke et al. [37], who found that contamination with trace metals such as Ga, In, Hg and Ru significantly reduced the growth of cowpea roots and caused cell rupture. This phytotoxic effect could be explained by the high binding tendency of these elements to the cell wall, increasing the cell rigidity, delaying the cell growth and finally causing the rupturing of cells [33]. It is important to recognize that the biogeochemical behavior of toxic elements like Zr in the environment and their phytotoxic impact on plants are primarily contingent on their speciation. Speciation refers to the existence of metals in diverse chemical forms, which is greatly influenced by environmental factors such as soil pH and interactions with soil organic matter [6]. Regarding Zr, it exists in the soil in different chemical forms that have a wide variety of solubility and bioavailability [38]. This speciation could explain the adverse effect of Zr upon plant growth and biomass. 

On the other hand, our study revealed that the phytotoxic effect of Zr was obviously mitigated by the application of eCO_2_. This ameliorative effect was embodied in the recovery of the plant biomass and photosynthetic efficiency relative to contaminated plants. In line with our findings, the growth of both maize and barley contaminated with As_2_O_3_ and HgO, respectively, exhibited a noticeable recovery in both growth and photosynthetic efficiency in response to treatment with eCO_2_ [39]. This advantageous effect might be ascribed to the ability of eCO_2_, within the physiological range, to enhance plant biomass by stimulating its photosynthetic machinery [33]. This, in turn, will modulate the photosynthetic carbon metabolism and so improve carbohydrate partitioning [10]. Furthermore, the enhancement in photosynthesis due to eCO_2_ can also be attributed to the elevated levels of CO_2_ at the RuBisco site, which reduces the availability of NADPH and ATP for photorespiration, thus facilitating CO_2_ assimilation in plants [40]. Kaiser et al. [41] reported that eCO_2_ increased the relative carbon gain with concomitant enhancements in the photosynthesis of tomato by initiating carboxylation reaction and slowing oxygenation reaction at RuBisco. Likewise, the photosynthesis in lettuce was improved in response to eCO_2_, the thing that reflected on its growth due to the availability of the carbon skeleton, resulting in higher levels of carbohydrates [42]. In conclusion, it is hypothesized that eCO_2_ could provide protection to significant crops against various environmental threats, including metal pollution like Zr, by enhancing their growth and photosynthesis. 

### 4.2. eCO_2_ Highly Quenched the Oxidative Damage Caused by Zr in Oat Plants

Improvement in plant growth and photosynthesis is associated with modulating the plant redox homeostasis, including the regulation of reactive oxygen species under various environmental cues [33]. Our results declared that Zr triggered oxidative stress in oat plants by increasing the levels of H_2_O_2_ and MDA. In this regard, the treatment of wheat seedlings with Zr caused an oxidative damage by altering the activities of the antioxidant enzymes [9]. Similarly, *Urtica dioica* grown in heavy-metal-contaminated soils experienced remarkable oxidative stress with concomitant DNA damage [43]. Likewise, soil contamination with Zn and Pd provoked noticeable oxidative damage in sorghum plants [44]. Oxidative stress was triggered in both wheat and soybean plants treated with As [45]. Moreover, cereal crops treated with tungsten nanoparticles exhibited a remarkable accumulation in both H_2_O_2_ and MDA, the main cause of oxidative damage in plants [46]. Plants respond to metal stress by triggering diverse signaling pathways, including those related to ROS metabolism, as tools to cope with the different environmental challenges [47]. The adverse effects of toxic metals, one of the environmental challenges, on the growth and oxidative status of plants could be due to direct or indirect reasons. The direct toxic impact lies in the ability of heavy metals to inhibit the cytoplasmic enzymes and the destruction of cell structures in response to oxidative damage [48]. Conversely, toxic metals can indirectly influence plant growth and oxidative balance by inhibiting the functions of soil microorganisms. This, in turn, impacts the decomposition of organic matter, leading to adverse effects on soil nutrient levels [49]. Furthermore, essential metabolic enzymes may be hampered due to the interference of the toxic metals with the soil microorganisms [50]. Toxic metals may also accumulate H_2_O_2_ by impairing photorespiration, one of most important H_2_O_2_-generating pools in plants [51]. Additionally, toxic metals caused ABA accumulation, leading to the hyperaccumulation of H_2_O_2_ and the slowing of cell division [52]. These detrimental effects, when combined, can impede plant growth and initiate plant oxidative damage. On the other hand, the co-application of eCO_2_ with Zr obviously reduced the oxidative damage caused by Zr alone by reducing the levels of H_2_O_2_ and MDA in oat plants. Similar results were obtained by AbdElgawad et al. [53], who proclaimed that the growth of both wheat and soybean in an atmosphere enriched with CO_2_ reduced the oxidative damage caused by As stress. The authors also reported that eCO_2_ enhanced the antioxidative defense system in cereals grown in As-contaminated soils [45]. Moreover, eCO_2_ obviously quenched the oxidative damage triggered in both wheat and maize treated with both NiO and HgO by diminishing the accumulation of H_2_O_2_ and MDA [13,54]. This mitigative impact of eCO_2_ could be attributed to its potency to reduce the chance of oxygenation reaction on RuBisco [55]. This will increase the rate of carboxylation reaction that, in turn, will increase the carbon assimilation [11]. Moreover, high levels of CO_2_ can reduce the activity of the enzymes of photorespiration, leading to a reduction in the accumulated H_2_O_2_, accordingly [56]. 

### 4.3. eCO_2_ Raised Oat Tolerance to Zr Contamination by Regulating the Antioxidant Defense System

In plants, the antioxidant defense system includes low molecular weight non-enzymatic antioxidants (ascorbate, glutathione, polyphenols, flavonoids, etc.) and antioxidant enzymes that function in a coordinated manner to restrain the over-production of ROS in plants [57]. Superoxide dismutase (SOD) is the key enzyme in the conversion of superoxide ions into H_2_O_2_, which is further turned into H_2_O by CAT, GPX, or APX [34]. Therefore, the elevation in the activities of such enzymes in response to Zr treatments is an attempt by the plant to rein in the overproduction of H_2_O_2_, one of the main causes of oxidative damage. We also opined an elevation in the ascorbate/glutathione pathway that functions mainly in scavenging H_2_O_2_ in oat plants under contamination conditions [58]. Pollution with Zr, like other environmental stresses, causes oxidative damage in oat plants. Likewise, we previously found that tungsten nanoparticles caused an increment in the components of both enzymatic and non-enzymatic antioxidants [46]. Moreover, the growth of Camellia sinensis in media containing Cd enhanced the accumulation of phenolics including flavans [59]. Similarly, oilseed rape treated with Cd showed a noticeable increase in the levels of non-enzymatic antioxidants [60]. Also, the application of Zn caused a noticeable increase in lipid peroxidation in *Brassica juncea* due to the hyperaccumulation of ROS [61]. Analogous findings were documented in the moss *Taxithelium nepalense*, wherein exposure to Cr and Pd led to the generation of reactive oxygen species (ROS), accompanied by a simultaneous reduction in the antioxidant defense system [62]. In rice, the application of Cu caused an over-accumulation of ROS and an observable decline in the antioxidant defense arsenal [63]. In this context, ascorbic acid was induced in wheat shoots subjected to nickel stress, indicating its pivotal role in ROS manipulation [64]. Moreover, the redox statuses of ASC (ASA/DHA) and GSH (GSH/GSSG) in oat plants were reduced in response to Zr contamination, the thing that delayed the antioxidant defense system in oat plants. Overall, plants activate the production of secondary metabolites such as phenylpropanoids in response to different environmental cues [65]. This process provides the cell with a wide array of phenolic metabolites by activating the phenylpropanoid pathway [66]. By activating the phenylpropanoid pathway, non-enzymatic antioxidants were synthesized to enhance plant tolerance against environmental stresses such as toxic metals [67].

On the other hand, eCO_2_ not only augmented the detoxification of ROS, but also kept the balance of both GSH/GSSG and ASC/DHA in Zr-polluted oat plants. In line with our findings, AbdElgawad et al. [68] declared that eCO_2_ caused a remarkable improvement in the growth of cereals grown in soils contaminated with As via augmenting the ascorbate/glutathione metabolism. It is worth noting that the ASC/DHA metabolic pool keeps the redox homeostasis in the photosystem by maintaining high ratios of GSH/GSSG and ASC/DHA, which is important for plant tolerance against different environmental challenges [69]. Therefore, the concurrent suppression of photorespiration and improvement of photosynthesis highlights the crucial contribution of eCO_2_ in mitigating the phytotoxic effect of Zr-contaminated oats. In other words, the mitigative role of eCO_2_ could be mainly due to its ability to increase carboxylation at the expense of the oxygenation reaction in RuBisco [10]. More interestingly, eCO_2_ can provide the cells with the C skeleton and energy needed for the growth of stressed plants [70]. This increment in carbon availability will supply the cells with antioxidant molecules, so raising the plant tolerance against the stress-induced oxidative damage [40]. In our study, the observed elevation in total antioxidant capacity (FRAP) due to treatment with eCO_2_ indicates an awakening within the plant to tolerate the ROS generated by Zr. In accordance, we opined a noticeable increment in the levels of polyphenols and flavonoids in response to eCO_2_ in plants contaminated with Zr (Figure 4). Indeed, this increment could quench the toxicity induced by Zr pollution as an adaptive response to environmental stresses [71]. Another perspective is that the sugars accumulated in response to enhanced photosynthesis can scavenge free radicals generated by ROS leading to alleviation of the oxidative damage caused by environmental stresses [72]. To this end, our study, for the first time, shed light on the positive role of increasing atmospheric CO_2_ in modulating the ROS homeostasis in oat plants to cope with pollution with Zr.

## 5. Conclusions

This investigation improves our knowledge about how eCO_2_ differentially alleviates the phytotoxic effect of Zr on the growth, photosynthesis, and redox status of *Avena sativa* plants. Contamination with Zr greatly reduced the photosynthesis of oat plants with concomitant reduction in their biomass. Moreover, contamination with Zr triggered remarkable oxidative stress in oats. On the other hand, eCO_2_ significantly recovered the adverse effect of Zr on the biomass and redox status of oats. Furthermore, eCO_2_ modulated the redox homeostasis of oat plants by enhancing their photosynthetic efficiency. Accordingly, enhanced photosynthesis can improve the plant biomass under both clean and contaminated conditions. This beneficial effect of eCO_2_ extends beyond photosynthesis and biomass; it also mitigates the severe oxidative damage in oats by decreasing the levels of H_2_O_2_ and MDA induced by Zr pollution. Moreover, eCO_2_ ameliorated the negative impact of Zr upon the non-enzymatic antioxidants by improving the total antioxidant capacity (FRAP) and then the levels of the non-enzymatic antioxidants (polyphenols and flavonoids). Moreover, eCO_2_ caused a noticeable activation in all measured antioxidant enzymes that were inhibited in oats contaminated with Zr. In conclusion, our study, for the first time, introduces a new insight on the ability of eCO_2_ in harnessing the redox homeostasis of oat plants to cope with the phytotoxic effect imposed by toxic metals, particularly Zr.

## Figures and Tables

**Table 1:** Two-way ANOVA on the significance of interaction between the two independent variables (Zr and eCO_2_) upon Zr content and the biomass and photosynthesis in oat plants (ns = non-significant; * = *p* < 0.05; ** = *p* < 0.01; *** = *p* < 0.001).

Dependent Variables	Type III Sum of Squares	df	Mean Square	F	Sig.
Zr content	178.230	1	178.230	51.630	***
FW	0.152	1	0.152	31.423	***
DW	0.002	1	0.002	8.790	*
Photosynthesis	0.194	1	0.194	0.231	ns
CHLa	0.000	1	0.000	4.014	ns
CHLb	0.000	1	0.000	6.258	*
CHLab	0.000	1	0.000	5.429	**
Carotenoids	0.000	1	0.000	0.958	ns
gs	0.005	1	0.005	0.001	ns
CHLflourec	0.009	1	0.009	80.172	***
Rubisco	7.279	1	7.279	0.989	ns

**Table 2:** Two-way ANOVA for the significance of interaction between the two independent variables (Zr and eCO_2_) upon oxidative damage, as well as the enzymatic and non-enzymatic antioxidants in oat plants (ns = non-significant; * = *p* < 0.05; ** = *p* < 0.01; *** = *p* < 0.001).

Dependent Variables	Type III Sum of Squares	df	Mean Square	F	Sig.
H_2_O_2_	5903.0	1	5903.0	12.62	*
MDA	4.114	1	4.114	48.04	***
FRAP	18.639	1	18.639	6.267	*
Polyphenol	3.157	1	3.157	2.338	***
Flavo	0.030	1	0.030	0.002	*
POX	0.226	1	0.226	10.63	*
CAT	.001	1	0.001	4.171	ns
SOD	1428.6	1	1428.6	37.0	***
APX	0.000	1	0.000	.000	ns
DHAR	0.217	1	0.217	14.90	**
MDHAR	0.000	1	0.000	4.004	ns
GR	0.006	1	0.006	1.939	ns
GPX	0.044	1	0.044	5.253	*

**Table 3:** Two-way ANOVA for the significance of interaction between the two independent variables (Zr and eCO_2_) upon ascorbate/glutathione metabolic pool in oat plants (ns = non-significant; * = *p* < 0.05; ** = *p* < 0.01; *** = *p* < 0.001).

Dependent Variables	Type III Sum of Squares	df	Mean Square	F	Sig.
ASC	0.000	1	0.000	0.159	ns
TASC	0.000	1	0.000	0.551	ns
DHA	0.000	1	0.000	9.152	*
ASC_DHA	0.163	1	0.163	36.236	***
GSH	0.000	1	0.000	9.543	**
TGSH	0.027	1	0.027	81.929	***
GSSG	0.020	1	0.020	131.102	***
GSH_GSSG	0.038	1	0.038	42.503	***

## Data Availability

Data presented in this study is included in this article.
